# Dynamical Tangles in Third-Order Oscillator with Single Jump Function

**DOI:** 10.1155/2014/239407

**Published:** 2014-12-03

**Authors:** Jiří Petržela, Tomas Gotthans, Milan Guzan

**Affiliations:** ^1^Department of Radio Electronics, Brno University of Technology, Purkynova 118, 612 00 Brno, Czech Republic; ^2^Department of Theoretical Electrotechnics and Electrical Measurement, Technical University of Kosice, Letna 9, 042 00 Kosice, Slovakia

## Abstract

This contribution brings a deep and detailed study of the dynamical behavior associated with nonlinear oscillator described by a single third-order differential equation with scalar jump nonlinearity. The relative primitive geometry of the vector field allows making an exhaustive numerical analysis of its possible solutions, visualizations of the invariant manifolds, and basins of attraction as well as proving the existence of chaotic motion by using the concept of both Shilnikov theorems. The aim of this paper is also to complete, carry out and link the previous works on simple Newtonian dynamics, and answer the question how individual types of the phenomenon evolve with time via understandable notes.

## 1. Introduction

It is well known that a majority of the real physical systems can be modeled by the system of the first-order differential equations with some sort of nonlinearity. In the case of the systems with at least three degrees of freedom the solution is not restricted to stable equilibrium or limit cycles but there is a certain chance to observe a much more complicated motion like chaos or hyperchaos [1]. This is a long-term unpredictable behavior caused by the so-called folding and stretching mechanism; first is responsible for solution bounded in finite state space volume and second for extreme sensitivity to the tiny changes of the initial conditions. Looking at this signal in time and frequency domain it resembles noise in many aspects. In reality, the individual waveforms combined together give rise to the strange attractors with fractal dimension [2] characterized by density, ergodicity, and mixing property. For chaotic attractor produced by third-order dynamical system value of geometrical dimension belongs to the range between two and three.

Since chaos is a robust steady state dynamical motion, it should be somehow distinguished from chaotic transients [3]. The rigorous mathematical tool proving its existence can be picked as one of the famous Shilnikov theorems (ST) [4]. Roughly speaking, if there hold certain conditions for the eigenvalues and the strategic orbits associated with the same equilibrium is discovered, the so-called Shilnikov's chaos can be observed. As will be clarified later, additional information must be obtained before the start of the searching procedure, such as location of the fixed points, eigenspaces, boundary planes, attraction sets, and corresponding basins. The description of procedure solving this problem for the famous Chuas equations [5] can be found in publication [6]. Many associated problems like vector field geometry of the so-called double-hook or dual double-scroll attractors [7] are solved in the interesting book [8]. Also chaos evolution principles for simple driven systems can be found here.

This paper is organized as follows. The second section introduces the mathematical model of the nonlinear oscillator and brings its brief linear analysis. The third section focuses on linear topological conjugacy LTC [9] and presents equivalent dynamical systems. In other words the question if the mathematical model under inspection forms an entire class of the dynamical systems will be answered using similar approach as demonstrated in [10]. The fourth section exhibits one possible approach to find two mirrored homoclinic orbits or heteroclinic connection between two fixed points. These trajectories are confirmed numerically together with associated chaotic behavior. The visualization of the basins of attraction and different manifolds is the core of the next section. Illustration of the structural stability of the chaotic attractors [11] by calculation of the largest Lyapunov exponents (LE) in the neighborhood of the nominal system parameters is a content of a next section. Such form of stability is essential from the viewpoint of physical construction of chaotic oscillator, for example, as electronic circuit. Since values of the circuit elements are functions of mathematical model parameters the sensitivity with respect to chaos deformation or destruction will be calculated. Finally concluding remarks, further research suggestions, and future topics are provided.

## 2. Mathematical Model

Assume the following dynamical system [12, 13] described by a single third-order differential equation, where the individual state variables can be interpreted as position, velocity, and acceleration, which belongs to the task from classical Newtonian dynamics:

(1) $$\begin{array}{c} \dddot{x}+\varphi_{1}\ddot{x}+\varphi_{2}\dot{x}=\xi_{1}x+\xi_{2}\mathrm{sign}\left(x\right),\\ \varphi_{1}^{A}=0.6,\;\;\varphi_{2}^{A}=1,\;\;\varphi_{1}^{B}=0.7,\;\;\varphi_{2}^{B}=0.8, \end{array}$$

where dots represent derivatives with respect to the independent variable, time. There are, in fact, two different dynamical systems, one for each sign combination inside right-hand side function. Let these variants denote by symbols *A*, *B* and let the nominal set of the parameters lead to the strange attractors:

(2) $$\begin{array}{c} \xi_{1}^{A}=-1.2,\;\;\xi_{2}^{A}=2,\;\;\xi_{1}^{B}=1.2,\;\;\xi_{2}^{B}=-0.6. \end{array}$$

Note that there is a significant degree of vector field symmetry. Both systems have simply defined boundary plane BP : {*x* = 0,  *y* ∈ *R* ^1^,  *z* ∈ *R* ^1^} separating the vector field into two outer affine segments and single virtual inner region. In further text, these segments will be denoted as *D*_+_, *D*_0_, and *D*_−_. There are just three equilibriums, one per each vector field segment, located at

(3) $$\begin{array}{c} x_{e0}={\left(\begin{array}{ccc} 0 & 0 & 0 \end{array}\right)}^{T},\;\;x_{\pm }={\left(\begin{array}{ccc} \pm \frac{\xi_{2}}{\xi_{1}} & 0 & 0 \end{array}\right)}^{T}. \end{array}$$

It is evident that the characteristic polynomial and set of the eigenvalues is the same for both fixed points. To be more specific it is cubic polynomial:

(4) $$\begin{array}{c} \lambda^{3}+\varphi_{1}\lambda^{2}+\varphi_{2}\lambda \mp \xi_{1}=\overset{3}{\underset{i=1}{\prod }}\left(\lambda -\lambda_{i}\right)=0. \end{array}$$

For the system case* A*, after substituting (2) in (1) we get a pair of the complex conjugated and a single real eigenvalue, namely, configuration *R* ^3^ ∈ *E*_1_^*s*^ ⊕ *E*_2_^*u*^:

(5) $$\begin{array}{c} \lambda_{1,2}=\lambda^{\prime }\pm \lambda^{\prime \prime }j=0.162\pm 1.128j,\;\;\lambda_{3}=-0.924. \end{array}$$

Thus fixed point is saddle-focus with stability index one. The vector field geometry resembles double scroll attractor generated by well-known Chua's equation with suppressed inner segment [5].

The system case *B* for the substitution (3) in (1) has the reverse stability index two, in detail *R* ^3^ ∈ *E*_1_^*u*^ ⊕ *E*_2_^*s*^:

(6) $$\begin{array}{c} \lambda_{1,2}=\lambda^{\prime }\pm \lambda^{\prime \prime }j=-0.594\pm 1.160j,\;\;\lambda_{3}=0.588. \end{array}$$

The fixed point located at the origin cannot be treated as a regular one; it acts more likely as a virtual equilibrium. The inner segment *D*_0_ is very narrow and cannot be easily observed even in the case of conventional numerical analysis. Following the rules of linear algebra we cannot derive additional useful information about dynamical system global motion. That is why further analysis is restricted to the existing numerical methods, that is, approaches which utilize the numerical integration process.

## 3. Linear Transform of Coordinates

Suppose original dynamical system (1)  $\dot{x}$ and associated system after linear transformation of the coordinates $\dot{\tilde{x}}$ written in the compact matrix form [14]

(7) $$\begin{array}{c} \dot{x}=Ax+b\mathrm{sign}\left(w^{T}x\right),\\ \dot{\tilde{x}}=T^{-1}AT\tilde{x}+T^{-1}b\mathrm{sign}\left(w^{T}T\tilde{x}\right), \end{array}$$

where **T** is regular square matrix (3 × 3) of the real numbers. Note that such transforms can shift, rotate, or linearly stretch and compress the state space volume in some direction while leaving eigenvalues unchanged. New system can be advantageous from the viewpoint of circuitry implementation, symbolic analysis, understanding underlying dynamics, attractor visualization, and so forth. Since (1) expressed in terms of (7) is already in normal form

(8) $$\begin{array}{c} A_{R}=\left(\begin{array}{ccc} 0 & 1 & 0\\ 0 & 0 & 1\\ \xi_{1} & -\varphi_{2} & -\varphi_{1} \end{array}\right),\;\;b_{R}=\left(\begin{array}{c} 0\\ 0\\ \xi_{2} \end{array}\right),\;\;w_{R}=\left(\begin{array}{c} 0\\ 0\\ 1 \end{array}\right), \end{array}$$

first example of LTC is the generation of state matrix **A**_*J*_ in Jordan form [15]. This is fundamental conversion with transformation matrix **T**_*J*_ with columns composed of the real and imaginary part of the complex eigenvector *λ*_*c*_ and real eigenvector *λ*_*r*_:

(9) TJ=Re(λc1)Im⁡(λc1)λr1Re(λc2)Im⁡(λc2)λr2Re(λc3)Im⁡(λc3)λr3,TJ−1ARTJ=AJ=λ′λ′′0−λ′′λ′000λ3,

where state matrix **A**_*R*_ directly represents system (1) and upper index of *λ*_*c*_ and *λ*_*r*_ denotes the corresponding element of the eigenvector. The knowledge of such system can be useful for motion analysis and return map diagnosis since eigenspaces in each region of the state space are orthogonal. Using the concept of LTC the concrete form of the state matrix **A** and vector **w** can be prescribed and transformation between desired system and equivalent system in the normal form can be established accordingly to [16]. The only restriction is that the resulting transformation must be regular square matrix. For the first equivalent system in the sense [17] the transformation **T**_*I*_ is

(10) $$\begin{array}{c} A_{I}=\left(\begin{array}{ccc} -\varphi_{1} & -1 & 0\\ \varphi_{2} & 0 & -1\\ -\xi_{1} & 0 & 0 \end{array}\right),\;\;w_{I}=\left(\begin{array}{c} 1\\ 0\\ 0 \end{array}\right),\\ T_{I}=\left(\begin{array}{c} w_{I}^{T}\\ w_{I}^{T}A_{I}\\ w_{I}^{T}A_{I}^{2} \end{array}\right)=\left(\begin{array}{ccc} 1 & 0 & 0\\ -\varphi_{1} & -1 & 0\\ \varphi_{1}^{2}-\varphi_{2} & \varphi_{1} & 1 \end{array}\right). \end{array}$$

Analogically for the second equivalent system the matrix **T**_*II*_ becomes

(11) $$\begin{array}{c} A_{II}=\left(\begin{array}{ccc} -\varphi_{1} & \varphi_{2} & -\xi_{1}\\ -1 & 0 & 0\\ 0 & -1 & 0 \end{array}\right),\;\;w_{II}=\left(\begin{array}{c} 1\\ 0\\ 0 \end{array}\right),\\ T_{II}=\left(\begin{array}{c} w_{II}^{T}\\ w_{II}^{T}A_{II}\\ w_{II}^{T}A_{II}^{2} \end{array}\right)=\left(\begin{array}{ccc} 1 & 0 & 0\\ -\varphi_{1} & \varphi_{2} & -\xi_{1}\\ \varphi_{1}^{2}-\varphi_{2} & -\varphi_{1}\varphi_{2}+\xi_{1} & \varphi_{1}\xi_{1} \end{array}\right). \end{array}$$

**Figure 1:**
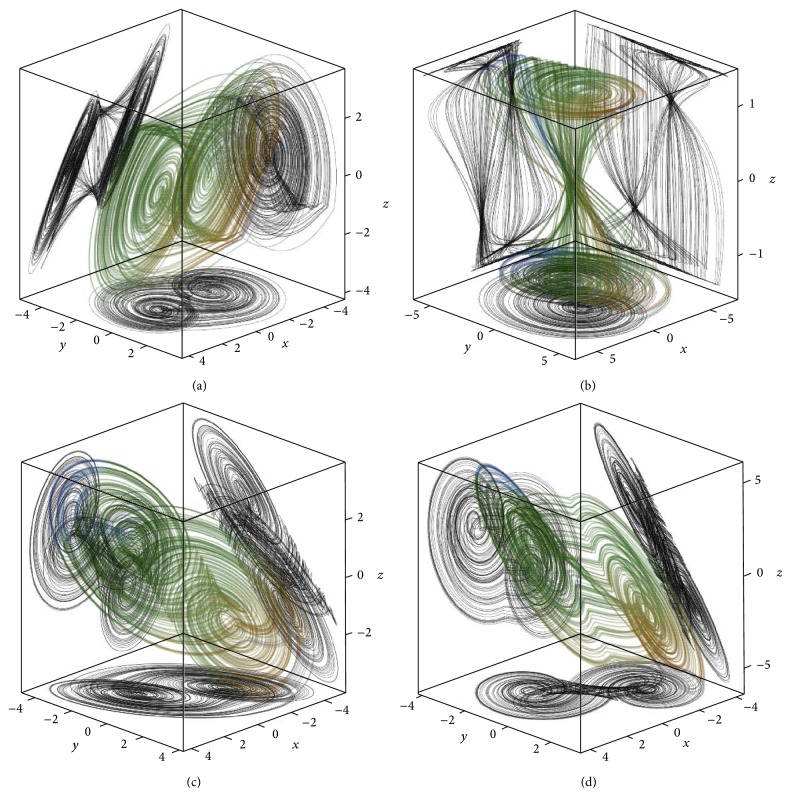
System configuration *A* in normal form (a), Jordan form (b), and first and second equivalent.

**Figure 2:**
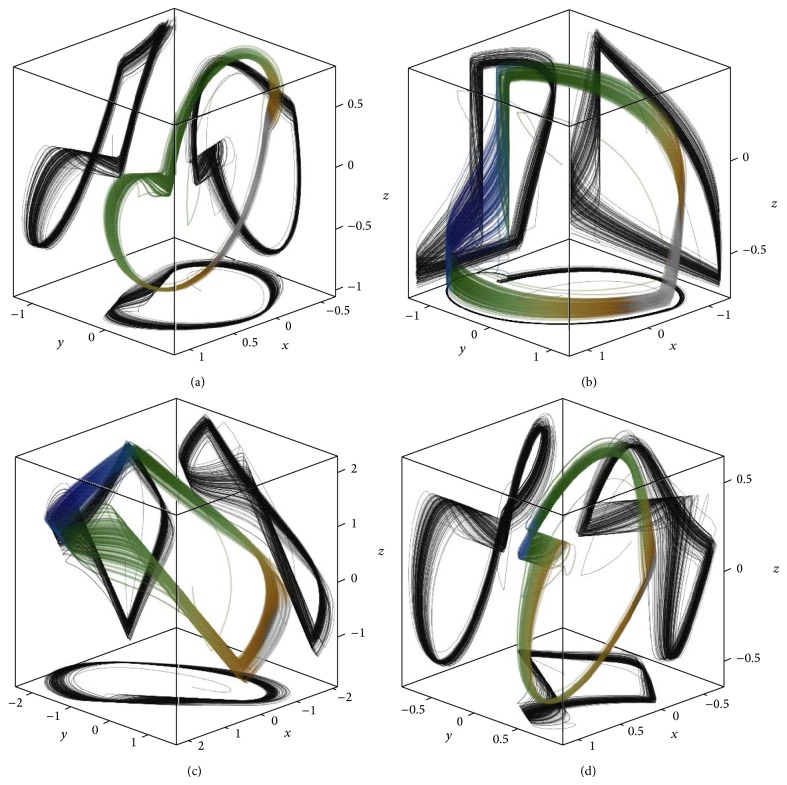
System configuration *B* in normal form (a), Jordan form (b), and first and second equivalent.

**Figure 3:**
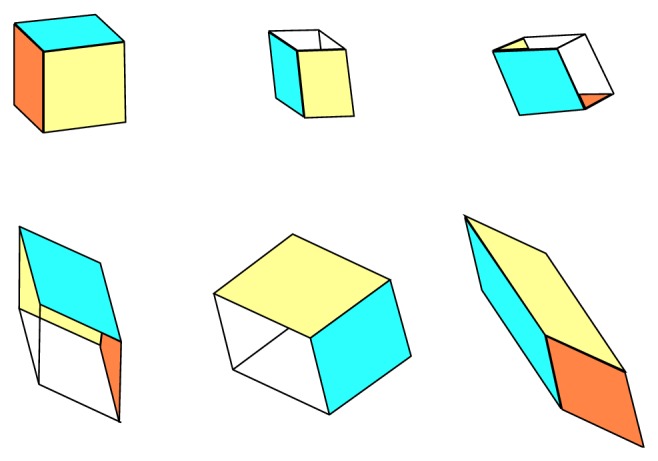
Geometrical visualization of LTC between equivalent systems; see text.

The three-dimensional perspective views on the chaotic state space attractors together with plane projections for each dynamical system mentioned above and obtained by using Mathcad with build-in fourth-order Runge-Kutta numerical integration method are shown in Figures 1 and 2, respectively. For these simulations final time equals *t*_max⁡_ = 1000 with time step *t*_step_ = 0.01. The initial conditions were set: **x**_0*A*_ = (0.1,0, 0)^*T*^ and **x**_0*B*_ = (0.1,0, 0)^*T*^. Note that LTC operation is demonstrated by means of Figure 3.

## 4. Strategic Trajectories

Before starting with description of the procedure for finding some strategic orbit in the sense of Shilnikov, the results (5) and (6) prompted that both essential conditions desired by ST are satisfied simultaneously for system of classes *A* and *B*:

(12) $$\begin{array}{c} \lambda^{\prime }\lambda_{3}<0\wedge \left|\lambda_{3}\right|>\left|\lambda^{\prime }\right|. \end{array}$$

Remember that this condition itself does not guarantee the presence of chaotic behavior; ST requires also strategic orbit associated with some fixed point. The problem with searching for specific state trajectory can be effectively converted into optimization task [18]. The basic form of the vector field with de facto two linear segments significantly simplifies the algebraic set of the fitness functions for optimization. The process of derivation of such set is described in step-by-step manner in paper [19]. The optimization routine seeks through parameter space, alters the eigenvalues, and rotates the corresponding eigenspaces simultaneously.

**Figure 4:**
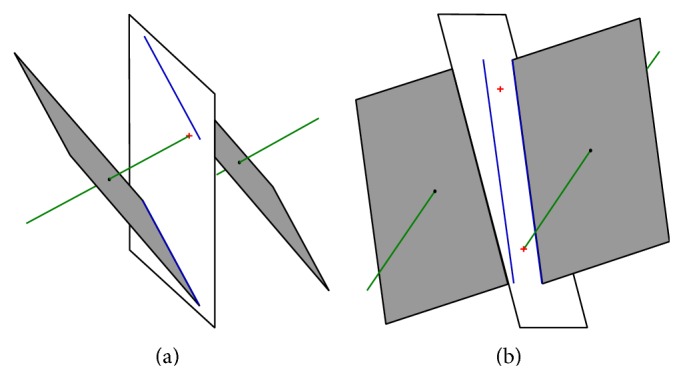
The geometric structures important for optimization, starting situation, and systems *A* (a) and *B* (b), where boundary planes are white, fixed points are black dots, eigenvectors are green, eigenplanes are gray, intersections of eigenvectors and boundary planes are red crosses, and intersections of eigenplanes and boundary plane are blue lines.

The goal function value is minimized and penalized if geometry of the vector field or desired property of the system changes. It is preserved by choosing the suitable guess values as well as by the restrictions on the parameter space under inspection. The geometric structures like points, lines, and planes important for optimization are defined in Figure 4.

### 4.1. Homoclinic Tangle

By definition, the homoclinic orbit is forwards and backwards asymptotic to the same equilibrium point. Due to the vector filed symmetry there is always a pair of the homoclinic tangles. Therefore we can focus on homoclinic orbit associated with fixed point **x**_−_ located in segment *D*_−_.

Following full integration method described in [20] tends to be very time consuming and, as a part of an optimization routine, it can diverge even in the case if homoclinic orbit exists. Our problem should be addressed separately for dynamical system cases *A* and *B*.

In case *B* let further investigation be focused on homoclic orbit associated with fixed point **x**_−_. Since we have unstable eigenvector we can start with numerical integration in the intersection of this eigenvector and boundary plane. Dynamical flow became a part of optimization procedure which will be stopped as soon as trajectory leaves segment *D*_+_. This corresponds to the unique mapping which should be satisfied:

(13) $$\begin{array}{c} {\left(0,\lambda_{3}\frac{\xi_{2}}{\xi_{1}},\lambda_{3}^{2}\frac{\xi_{2}}{\xi_{1}}\right)}^{T}\overset{\Phi_{t>0}}{\to }{\left(0,\alpha ,\left(\frac{\xi_{2}}{\xi_{1}}-\omega_{1}\frac{\alpha -(\xi_{2}/\xi_{1})(\sigma_{2}/\sigma_{1})}{\omega_{2}-\omega_{1}(\sigma_{2}/\sigma_{1})}\right)\frac{\sigma_{3}}{\sigma_{1}}+\omega_{3}\frac{\alpha -(\xi_{2}/\xi_{1})(\sigma_{2}/\sigma_{1})}{\omega_{2}-\omega_{1}(\sigma_{2}/\sigma_{1})}\right)}^{T}, \end{array}$$

where *α* is arbitrary value.

In the case *A*, the situation is analogical, except that backward integration instead of standard is performed. To get homoclinic connection there must exist a mapping (13) but for times *t* < 0.

Final trajectory will consist of three pieces; free-motion in one segment of the vector field and forced motions along unstable eigenspace (forward integration) and stable eigenspace (backward integration) in the other segment. Utilizing this concept the new set of the parameters for system case *A* has been found as *φ*_1_ = 0.63339561, *φ*_2_ = 1.00676348, *ξ*_1_ = −1.29333691, *ξ*_2_ = 1.98006640, initial conditions **x**_0*A*_ = (−*ξ*_2_/*ξ*_1_, 0,0)^*T*^ + (1 · 10^−10^, 0,0)^*T*^, fourth order Runge-Kutta build-in MATLAB function integration with variable step (initial step *h* = 1 · 10^−6^) and with maximal step *h* = 1 · 10^−2^, and final value of fitness function 1.3 · 10^−3^.

**Figure 5:**
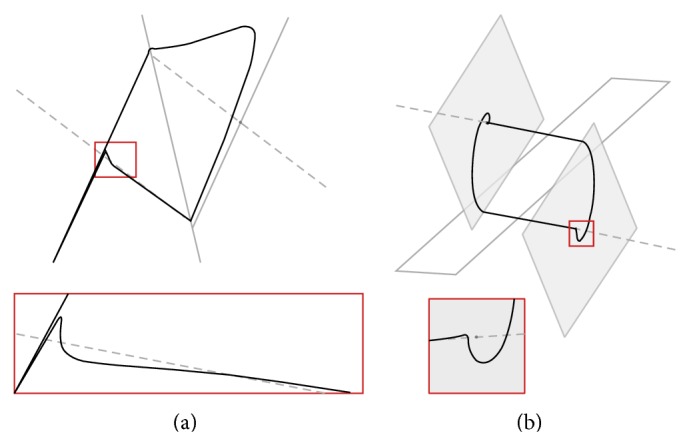
Trajectory close to the homoclinic orbit for dynamical system case *A* (a) and case *B* (b).

Similarly for dynamical system case B we get *φ*_1_ = 0.715499177650, *φ*_2_ = 0.577154986868, *ξ*_1_ = 1.284935534298, *ξ*_2_ = −0.631662627700, initial conditions **x**_0*B*_ = (*ξ*_2_/*ξ*_1_, 0,0)^*T*^ − (1 · 10^−8^, 0,0)^*T*^, fourth order Runge-Kutta build-in MATLAB function integration with variable step (initial step *h* = 1 · 10^−6^) and with maximal step *h* = 1 · 10^−2^, and final value of fitness function 7.62 · 10^−4^. The numerically integrated trajectories are provided in Figure 5.

A careful reader can raise an objection that integration as a part of optimization can be removed by solving a system of the linear differential equations. This is possible although the resulting analytical formulas are quite complicated. By considering known initial conditions and substituting desired line-type intersection it is possible to separate internal system parameters *φ*_1_, *φ*_2_, *ξ*_1_, and *ξ*_2_. Thus this approach also cannot provide a time required for trajectory to leave affine segment under inspection.

### 4.2. Heteroclinic Tangle

The heteroclinic orbit can be considered as a generalization of a saddle loop. In fact, searching for the heteroclinic connection is a two-dimensional problem. Since objective functions are known analytically MATLAB and build-in gradient-based technique has been utilized. The fitness function for this geometric structure is minimization of distance between point *X* and line *Y* such that

(14) z¯−ξ2ξ1−ω1y¯−(ξ2/ξ1)(σ2/σ1)ω2−ω1(σ2/σ1)σ3σ1  −ω3y¯−(ξ2/ξ1)(σ2/σ1)ω2−ω1(σ2/σ1)=0,

where **ω** is an imaginary part of the complex eigenvector and **σ** represents its real part and auxiliary constants. Although the analytic solution can be derived the time necessary for this unique mapping is unknown. Thus a numerical integration process should be a part of the optimization with the initial conditions set in vicinity of some fixed point.

For the first sign case of the differential equations (1) one can found the following set of the parameters: *φ*_1_ = −0.6261, *φ*_2_ = 0.9536, *ξ*_1_ = −1.2655, and *ξ*_2_ = 1.9937. Analogically for case *B* sign variant we get *φ*_1_ = −0.6378, *φ*_2_ = 0.6011, *ξ*_1_ = 1.3156, and *ξ*_2_ = −2.1697.

**Figure 6:**
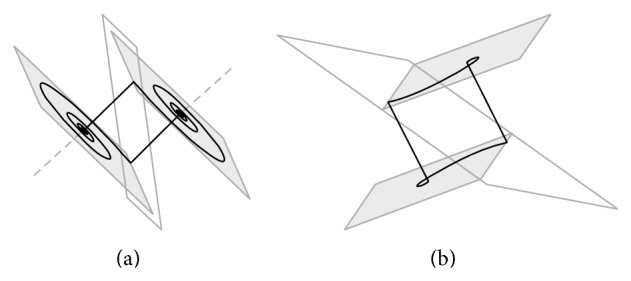
Trajectory close to the heteroclinic orbit for dynamical system cases *A* (a) and *B* (b).

The strategic orbits are unstable such that for numerical integration the initial conditions must be chosen carefully. For the strategic orbits, the process of numerical integration must begin in the close neighborhood of the fixed point. Using time-forward integration the state point goes into the direction of unstable manifold towards the opposite equilibrium. The saddle loop is finished by using time-backward numerical integration. The resulting state trajectories for both sets of the parameters are given in Figure 6. Despite being structurally unstable, these trajectories can be constructed and destructed via a manipulation with vector field geometry, that is, by changing the internal system parameters. Recently it has been verified that inverse approach can be used; starting with the satisfaction of one ST the original mathematical model can be derived [21].

## 5. Overall Numerical Analysis

Although studied dynamical system is algebraically quite simple, it is nonlinear and chaotic. Therefore there is no closed-form analytic solution. Thus the analysis is restricted to the numerical procedures mostly based on integration of the state space trajectory. This process is also involved in the routine for calculation of the spectrum of the LE [22]. These real numbers measure the average ratio of exponential separation between trajectories in the state space. For chaotic motion it is necessary to have one positive LE; the sum of all LE must be negative since the dynamics is dissipative due to the parameter *φ*_1_ > 0. This concept has been used to prove that the region for chaos is wide enough to preserve some structural stability [23] of the desired strange attractor; such form of stability allows developing the electronic circuits with the same dynamics [24]. The routine for LE spectrum computation can be effectively utilized also for the purpose of detecting chaos in the general class of arbitrary-order dynamical systems; for further details see [25]. Due to discontinuity in the system the derivations in Jacobi matrix have to be threated in order to determine Lyapunov exponents correctly. We obtained more precise results by using sigmoid function with extreme value of *γ* as defined in [26]. One other possible demonstration dealing with discontinuity in the forced system can be found in [27].

**Figure 7:**
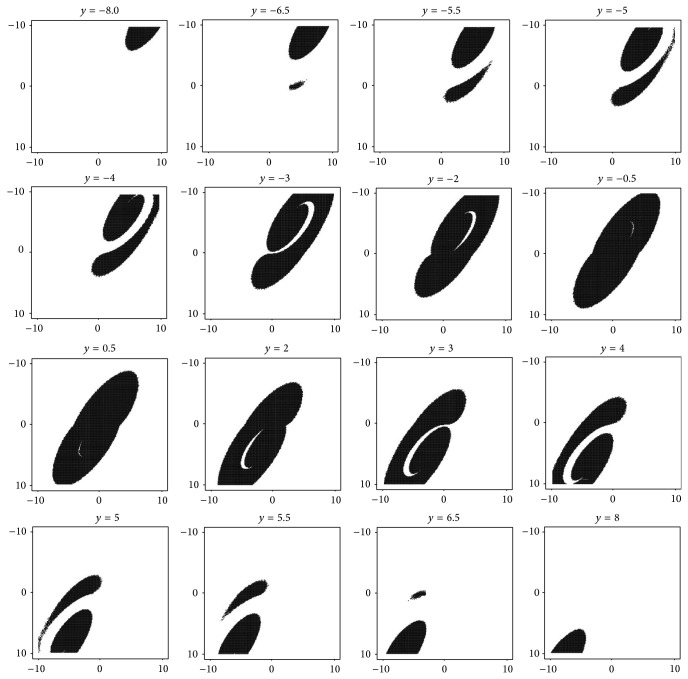
The graphical illustration of the basin of attraction for chaotic attractor, system case *A*. These slices are *x*-*z* projections of cross-sections with the *y*-axis. Black region represents initial condition inside basin of attraction. All other trajectories tend to infinity.

**Figure 8:**
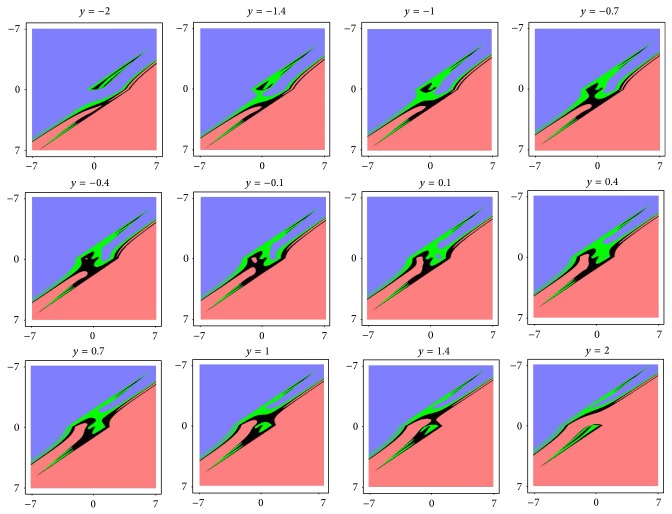
The graphical illustration of the basin of attraction for chaotic attractor, system case *B*. These slices are cross-sections of the *y*-axis where *y* = {−2, − 1.4, − 1, − 0.7, − 0.4, − 0.1, 0.1, 0.4, 0.7, 1, 1.4, 2} (left to right). Black and green regions mark basins of attraction for case *A* and case *B*, respectively. Blue and red regions denote unbounded solutions.

The remaining question which should be answered is the following: how many attractors are available for the nominal set of the parameters and show the associated basins of attractions. The most straightforward approach to visualize these subspaces is by means of repeated integrations. The grid for the initial condition was a cube with edge lengths *δ* ∈ (−2,2) with 400 points. Due to the symmetry of the vector field two mirrored strange attractors are highly expected. It eventually turns out that the system case *A* has only unbounded solution or chaotic attractor; trivial fixed point solution is out of question. These results are visible in Figure 7. For the system case *B* two chaotic attractors have been found; see Figure 8.

**Figure 9:**
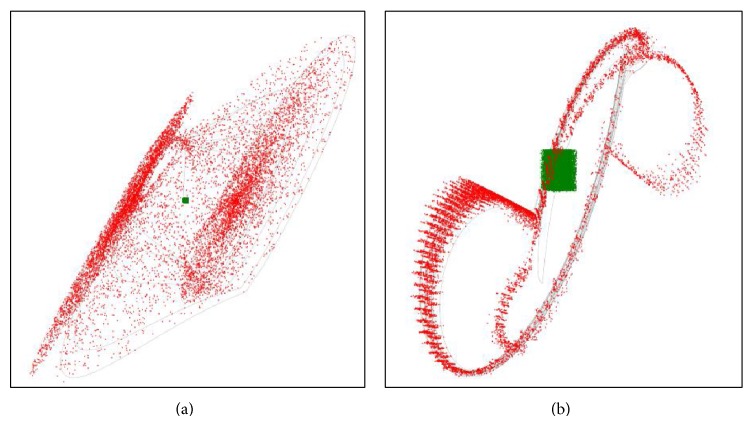
The graphical illustration of the sensitivity to initial conditions. Where the total number of random generations is 10 · 10^3^ with standard deviation *σ* = 0.01 around initial point ${\underline{x}}_{0AB}={\left(0,0,0\right)}^{T}$, total time *T*_end_ = 100, and integration step *h* = 0.01. On the left there is case A and on the right there is case *B*. The green points represent initial conditions, red color is a final point, and gray is original attractor.

The graphical illustration of the sensitivity to initial conditions can be seen in Figure 9. The total number of random generations is 10 · 10^3^ with standard deviation *σ* = 0.01 around initial point *x*_0*AB*_ = (0,0, 0)^*T*^, total time *T*_end_ = 100, and integration step *h* = 0.01.

## 6. Circuitry Implementation

**Figure 10:**
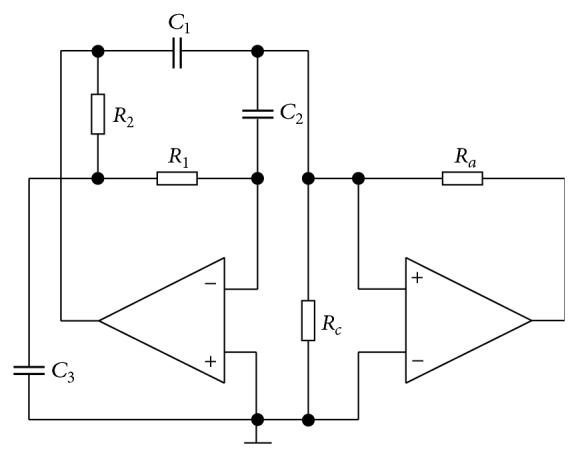
The circuitry implementation of chaotic oscillator for case *A*.

In order to evaluate geometrical structural stability of equations a new circuit (not presented so far for (1)) was assembled and measured. The circuit synthesis methods dedicated to modeling the nonlinear dynamical systems are well-known and commonly used [28, 29]. Assume canonical (in the sense of minimum circuit components) network shown in Figure 10 which represents a parallel connection of the third-order linear admittance and two-segment piecewise-linear resistor. The straightforward analysis gives us admittance function in Laplace transform, namely,

(15) Ys=s3+φ1s2φ2s≈c1c2c3r1r2s3+c1c2(r1+r2)s2+(c1+c2)s,

where resistors and capacitors have normalized values so far. To obtain the real passive element values let consider impedance normalization factor *ξ*_*I*_ and frequency norm *ξ*_*F*_. By comparing the individual coefficients (15) with (1) we get following simple relationships

(16) $$\begin{array}{c} C_{1}=C_{2}=\frac{\varphi_{2}}{2\xi_{I}\xi_{F}},\;\;C_{3}=\frac{\varphi_{2}^{2}}{\varphi_{1}^{2}\xi_{I}\xi_{F}},\;\;R_{1}=R_{2}=\frac{2\varphi_{1}\xi_{I}}{\varphi_{2}^{2}}. \end{array}$$

The amper-voltage characteristics of nonlinear resistor can be defined by two equations depending on the sign of input voltage, in detail:

(17) $$\begin{array}{c} I=f\left(V\right)=-\left(\frac{1}{r_{a}}+\frac{1}{r_{c}}\right)V+\frac{V_{\text{Sat}}}{r_{a}}\mathrm{sign}\left(V\right), \end{array}$$

where *V*_sat⁡_ ≈ 13 V represents a saturation voltage of the used operational amplifier TL082 and orientation of current is outwards the resistor. In fact note that due to saturation in practice the function has four segments. By adopting the numerical constants (2) and norms *ξ*_*I*_ = 10^4^ and *ξ*_*F*_ = 10^4^ we get the final values of the passive elements to generate double-scroll attractor:

(18) $$\begin{array}{c} C_{1}=C_{2}=5\;\text{nF},\;\;C_{3}=27\;\text{nF},\;\;R_{1}=R_{2}=12\;\text{k}\Omega ,\\ R_{a}=65\;\text{k}\Omega ,\;\;R_{c}=9.6\;\text{k}\Omega . \end{array}$$

**Figure 11:**
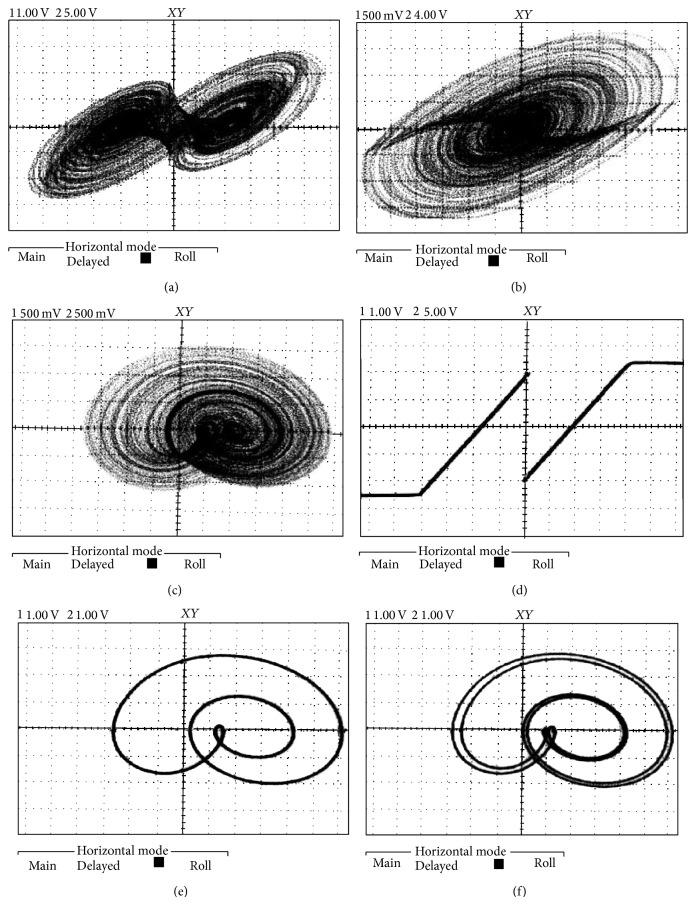
Several examples of the laboratory measurements (for case *A*): double-scroll attractor (a,b), single-scroll (c), AV curve of nonlinear resistor including saturation (d), limit cycle (e), and period doubling (f).

The experimental verification (measurements) of this chaotic oscillator is provided by means of Figure 11. Note that proposed circuitry represents only case *A* dynamical system (2). Variant *B* can be modeled by using slightly modified nonlinear resistor. Transformation from normal form into voltages across capacitors is represented by square regular matrix:

(19) $$\begin{array}{c} T\left(\begin{array}{c} x\\ y\\ z \end{array}\right)=\left(\begin{array}{c} u_{c_{1}}\\ u_{c_{2}}\\ u_{c_{3}} \end{array}\right),\;\;T=\left(\begin{array}{ccc} -1 & c_{2}\left(r_{1}+r_{2}\right) & c_{2}c_{3}r_{1}r_{2}\\ 1 & 0 & 0\\ 0 & c_{2}r_{1} & 0 \end{array}\right). \end{array}$$

**Figure 12:**
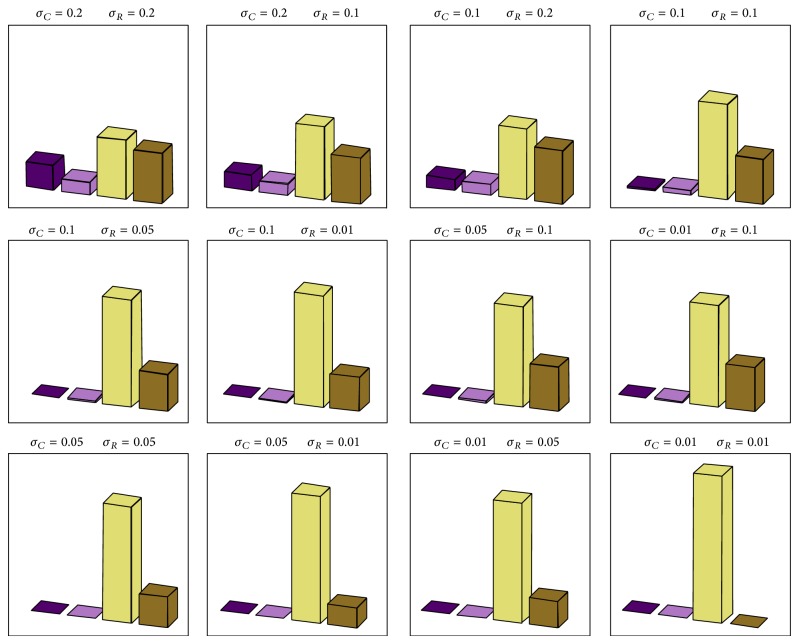
Monte Carlo analysis (for case *A*) for various standard deviations (Gaussian distributed) around nominal values (2) for resistors *σ*_*R*_ and capacitors *σ*_*C*_. Graphs can be understood as histograms in %, where the sum of values in histogram represents 100%. Dark magenta denotes fixed point solution, pink color is a limit cycle, yellow is chaotic motion, and brown color marks unbounded trajectories.

Figure 12 give an idea if uncertainties in values of the passive components are capable to destruct chaotic nature of circuit. It is obvious that 1% tolerances cannot cause such changes, but 10% tolerances can lead to death of double-scroll in about 8% of cases. The total number of turns for each histogram is 10000 (for case *A*) with initial conditions **i**_*c*_ = (0.1,0, 0)^*T*^.

## 7. Conclusion

This contribution addresses problems associated with complete analysis of the third-order dynamical system with single jump-type nonlinearity. The dynamical system under inspection belongs to the generalized class of the Chuas equations published in [26]. The existence of the Shilnikov chaos has been proved by using optimization and numerical integration. The specialty of given dynamical system is in changing the sign of nonlinear function; the homoclinic orbits or heteroclinic orbits appear. That has not been demonstrated yet. However the electronic circuit capable to model the nonlinear behavior near the strategic orbits has not been presented so far.

There are many unsolved topics involving dynamical systems with possible chaotic solution. A fraction of them are mentioned in the paper [30]; but personal experience and attempts to solve real specific system necessarily unfold many others. The universality of chaotic behavior is uncovered in [31] where several examples from different scientific fields are provided.

Achieved results can be generalized for multiscroll spiral attractors [7, 32].

The shape of the strange attractors generated by system cases *A* and *B* suggests that there are the same sources of chaos, perturbed heteroclinic orbit (double-scroll attractor).

## References

[1] Sprott J. C. (2003). *Chaos and Time-series Analysis*.

[2] Hentschel H. G. E., Procaccia I. (1983). The infinite number of generalized dimensions of fractals and strange attractors. *Physica D: Nonlinear Phenomena*.

[3] Gans R. F. (1995). When is cutting chaotic?. *Journal of Sound and Vibration*.

[4] Silva C. P. (1993). Shil'nikov's theorem—a tutorial. *IEEE Transactions on Circuits and Systems I: Fundamental Theory and Applications*.

[5] Chua L. O., Komuro M., Matsumoto T. (1986). The double scroll family. I. Rigorous proof of chaos. *IEEE Transactions on Circuits and Systems*.

[6] Spany V., Galajda P., Guzan M., Pivka L., Olej{\'a}r M. (2010). Chua's singularities: great miracle in circuit theory. *International Journal of Bifurcation and Chaos*.

[7] Piper J. R., Sprott J. C. (2010). Simple autonomous chaotic circuits. *IEEE Transactions on Circuits and Systems II: Express Briefs*.

[8] Kapitaniak T. (1992). *Chaotic Oscillators*.

[9] Wu C. W., Chua L. O. (1996). On linear topological conjugacy of Lur'e systems. *IEEE Transactions on Circuits and Systems. I. Fundamental Theory and Applications*.

[10] Pospisil J., Brzobohaty J., Kolka Z., Horska J. (1999). New canonical state models of Chua's circuit family. *Radioengineering*.

[11] Sprott J. C. (2000). Simple chaotic systems and circuits. *American Journal of Physics*.

[12] Sprott J. C. (2010). *Elegant Chaos: Algebraically Simple Chaotic Flows*.

[13] Sprott J. C. (2000). A new class of chaotic circuit. *Physics Letters A*.

[14] Kocarev L. M., Stojanovski T. D. (1996). Linear conjugacy of vector fields in Lur'e form. *IEEE Transactions on Circuits and Systems. I. Fundamental Theory and Applications*.

[15] Petrzela J. (2009). Three-segment piecewise-linear vector fields with orthogonal eigenspaces. *Acta Electrotechnica et Informatica*.

[16] Feldmann U., Schwarz W. (1994). Linear conjugacy of n-dimensional piecewise linear systems. *IEEE Transactions on Circuits and Systems I: Fundamental Theory and Applications*.

[17] Pospisil J., Kolka Z., Horska J., Brzobohaty J. (2000). Simplest ODE equivalents of Chua's equations. *International Journal of Bifurcation and Chaos*.

[18] Mees A. I., Chapman P. B. (1987). Homoclinic and heteroclinic orbits in the double scroll attractor. *IEEE Transactions on Circuits and Systems*.

[19] Petrzela J. (2010). On the strategic orbits in third-order oscillator with jump nonlinearity. *International Journal of Algebra*.

[20] Medrano T. R. O., Baptista M. S., Caldas I. L. (2003). Homoclinic orbits in a piecewise system and their relation with invariant sets. *Physica D: Nonlinear Phenomena*.

[21] Zhou T., Chen G., Yang Q. (2004). Constructing a new chaotic system based on the Shilnikov criterion. *Chaos, Solitons & Fractals*.

[22] Grygiel K., Szlachetka P. (1995). Lyapunov exponents analysis of autonomous and nonautonomous sets of ordinary differential equations. *Acta Physica Polonica B*.

[23] Petržela J., Hruboš Z., Gotthans T. (2011). Modeling deterministic chaos using electronic circuits. *Radioengineering*.

[24] Petrzela J., Kolka Z., Hanus S. (2006). Simple chaotic oscillator: from mathematical model to practical experiment. *Radioengineering*.

[25] Gotthans T., Petrzela J., Hrubos Z., Baudoin G. Parallel particle swarm optimization on chaotic solutions of dynamical systems.

[26] Brown R. (1993). Generalizations of the Chua equations. *IEEE Transactions on Circuits and Systems I: Fundamental Theory and Applications*.

[27] Grantham W. J., Lee B. (1993). A chaotic limit cycle paradox. *Dynamics and Control*.

[28] Itoh M. (2001). Synthesis of electronic circuits for simulating nonlinear dynamics. *International Journal of Bifurcation and Chaos in Applied Sciences and Engineering*.

[29] Elwakil A. S., Kennedy M. P. (2001). Construction of classes of circuit-independent chaotic oscillators using passive-only nonlinear devices. *IEEE Transactions on Circuits and Systems. I. Fundamental Theory and Applications*.

[30] Elhadj Z., Sprott J. C. (2011). Some open problems in chaos theory and dynamics. *International Journal of Open Problems in Computer Science and Mathematics*.

[31] Zeraoulia E. (2011). *Models and Applications of Chaos Theory in Modern Sciences*.

[32] Gotthans T., Hruboš Z. (2013). Multi grid chaotic attractors with discrete Jumps. *Journal of Electrical Engineering*.

